# CAR-T Cells for the Treatment of Lung Cancer

**DOI:** 10.3390/life12040561

**Published:** 2022-04-08

**Authors:** Luisa Chocarro, Hugo Arasanz, Leticia Fernández-Rubio, Ester Blanco, Miriam Echaide, Ana Bocanegra, Lucía Teijeira, Maider Garnica, Idoia Morilla, Maite Martínez-Aguillo, Sergio Piñeiro-Hermida, Pablo Ramos, Juan José Lasarte, Ruth Vera, Grazyna Kochan, David Escors

**Affiliations:** 1 Oncoimmunology Group, Navarrabiomed, Instituto de Investigación Sanitaria de Navarra (IdiSNA), 31008 Pamplona, Spain; luisa.chocarro.deerauso@navarra.es (L.C.); lfernanr@navarra.es (L.F.-R.); ester.blanco.palmeiro@navarra.es (E.B.); miriam.echaide.gorriz@navarra.es (M.E.); ai.bocanegra.gondan@navarra.es (A.B.); maider.garnica.suberviola@navarra.es (M.G.); sergio.pineiro.hermida@navarra.es (S.P.-H.); pablo.ramos.castellanos@navarra.es (P.R.); grazyna.kochan@navarra.es (G.K.); david.escors.murugarren@navarra.es (D.E.); 2 Medical Oncology Department, Hospital Universitario de Navarra, Instituto de Investigación Sanitaria de Navarra (IdiSNA), 31008 Pamplona, Spain; lucia.teijeira.sanchez@navarra.es (L.T.); idoia.morilla.ruiz@navarra.es (I.M.); maite.martinez.aguillo@navarra.es (M.M.-A.); ruth.vera.garcia@navarra.es (R.V.); 3 Division of Gene Therapy and Regulation of Gene Expression, Centro de Investigación Médica Aplicada (CIMA), Instituto de Investigación Sanitaria de Navarra (IdiSNA), 31008 Pamplona, Spain; 4 Immunology and Immunotherapy, Centro de Investigación Médica Aplicada (CIMA), Instituto de Investigación Sanitaria de Navarra (IdiSNA), 31008 Pamplona, Spain; jjlasarte@unav.es; 5 Oncobiona Group, Navarrabiomed, Instituto de Investigación Sanitaria de Navarra (IdiSNA), 31008 Pamplona, Spain

**Keywords:** CAR-T, adoptive cell therapy, immunotherapy, solid tumors, lung cancer

## Abstract

Adoptive cell therapy with genetically modified T lymphocytes that express chimeric antigen receptors (CAR-T) is one of the most promising advanced therapies for the treatment of cancer, with unprecedented outcomes in hematological malignancies. However, the efficacy of CAR-T cells in solid tumors is still very unsatisfactory, because of the strong immunosuppressive tumor microenvironment that hinders immune responses. The development of next-generation personalized CAR-T cells against solid tumors is a clinical necessity. The identification of therapeutic targets for new CAR-T therapies to increase the efficacy, survival, persistence, and safety in solid tumors remains a critical frontier in cancer immunotherapy. Here, we summarize basic, translational, and clinical results of CAR-T cell immunotherapies in lung cancer, from their molecular engineering and mechanistic studies to preclinical and clinical development.

## 1. Introduction

Immunotherapy has gained a prominent role in the treatment of cancer, especially immunotherapies based on immune checkpoint blockade and adoptive T cell transfer [1]. Treatment with immune checkpoint inhibitors (ICIs) consist of the systemic administration of antibodies blocking inhibitory interactions between cancer cells and T cells, and thus unleashing antitumor immune responses. These treatments can be administered as monotherapies or in combination with chemotherapies. The most widely used are based on antibodies that block PD-L1/PD-1 interactions [2,3,4,5]. ICI blockade strategies are effective but present some clinical problems: Many patients show primary or acquired resistance and there is a lack of reliable predictive biomarkers of response. In addition, these treatments have a relatively high cost and can induce inflammatory or autoimmune toxicities in some patients. Furthermore, an acceleration of tumor growth rate may occur after the administration of ICIs in a set of patients called hyperprogressive disease, which is associated with fast clinical deterioration [6,7,8,9].

Adoptive T cell immunotherapy, or therapeutic combinations of adoptive T cell transfer (ACT) with ICI blockade, might overcome the limitations of ICI monotherapies and improve the outcomes of some patients. ACT strategies comprise several modalities. In these immunotherapies, T cells are genetically engineered to specifically recognize and kill cancer cells through the expression of chimeric antigen receptors (CARs) or tumor antigen-specific T-cell receptors (TCRs) [10]. The most effective based on clinical relevance are those in which autologous T cells are modified with lentiviral and retroviral vectors to express CARs or TCRs [11,12,13,14,15].

CAR-T cells hold great promise as advanced and personalized therapies for the treatment of cancer. During recent years, several of these cellular products have been granted approval by the FDA for the treatment of hematological malignancies [16]. For CAR-T therapy on solid tumors, modest results have been published from several phase I/II trials. However, more data and further development is still required for the use of ACT for solid tumors.

## 2. Pre-Clinical Development of CAR-T Cells

**Figure 1:**
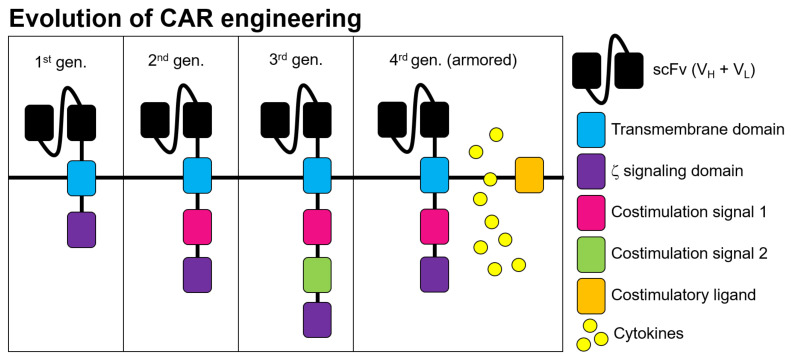
Evolution of CAR engineering. The scFv domain confers target antigen specificity. The transmembrane domain anchors the CAR to the T cell membrane. The 1st generation (gen.) CARs engineered contained a CD3ζ or FcRγ signaling domain, 2nd and 3rd gen. CARs have one or two costimulatory domains in series with the CD3ζ domain. The two most common costimulatory domains are CD28 and 4-1BB, both associated with high patient response rates. The 4th gen. CAR-T can be additionally engineered to release transgenic cytokines or its intracellular signaling can be induced by an external costimulatory ligand.

The chimeric antibody-T cell receptor molecules are generally structured as a single-chain immunoglobulin variable domain for extracellular binding to its target (scFvs), a hinge region, a transmembrane domain, and one or more intracellular signaling domains from T cell co-stimulatory molecules such as CD28 or 4-1BB (Figure 1) [17]. In 1989 Gross, Waks and Eshhar first described the design and expression of immunoglobulin-T-cell receptor chimeric molecules as functional receptors with antibody-type specificity [18]. The authors of the study expressed these chimeric genes fusing the TCR constant domains to the variable domains of an anti-2,4,6-trinitrophenyl (TNP) antibody (SP6). In 1993, the authors combined scFv antibody-binding domains and the gamma (γ) or zeta (ζ) chains of the CD3 molecules, demonstrating specific target recognition and cellular activation of cytotoxic lymphocytes [19]. This is considered the first first-generation CAR, although it was not clinically effective yet. 

Over the next decades, research on CARs increased leading to first, second, third, and currently fourth generation CAR designs, developed as part of an emerging new immuno-pharmacology field [17,20]. First generation CARs consist of the CD3ζ or CD3γ gamma chains alone, whereas second generation CARs added signaling domains from T cell co-stimulatory molecules such as CD28 or 4-1BB. The incorporation of these signaling domains recapitulate the proper activation of the TCR complex when engaged with the MHC-peptide and co-stimulatory receptors. Thus, the CAR molecule enhances T cell functions in terms of effector functions, proliferation, cytokine secretion, resistance to off-tumor toxicities such as apoptosis, efficacy, and in vivo persistence [17,21,22]. Third generation CARs boosted these effector functions, efficacy, and persistence even more by simultaneously introducing the signaling domains of CD28 and 4-1BB [23,24,25]. 

After 10 years of development from the first description of CARs, the first preclinical evidence of efficacy of tumor-reactive human CAR-T was described [26,27]. This specific CAR design targeted the B-cell-specific CD19 surface antigen. Co-stimulation was induced with CD80 and IL-15. This second generation CD19-targeted CAR demonstrated efficacious killing of chronic lymphocytic leukemia (CLL) cancer cells in in vitro and in vivo studies. 

Furthermore, fourth generation CARs are currently being developed, the so-called T cells redirected for universal cytokine-mediated killing (TRUCKs) or armored-CARs [28]. These CAR structures are designed to improve CAR-T proliferation and anti-tumor efficacy [29]. TRUCKs consist of CAR molecules that incorporate two or three signaling domains of co-stimulatory molecules such as CD28, 4-1BB, and CD40L, or they can be engineered to contain CAR-inducible transgenic cytokines such as IL-12, IL-17, or CCL19 to modulate the tumor microenvironment [17,23,30,31,32,33].

In addition to CAR design, the so-called “Smart CAR-T cell products” combine distinct strategies to improve their efficacy. For example, pooled CAR-T cell preparations, multi-CAR-T cells, tandem CAR-T cells, conditional CAR-T cells, split CARs, iCARs, or suicide switches, among others. Pooled CAR-T cells consist of a mixture of at least two single targeting engineered CAR-T cells, each of which has different antigen specificities. For example, Feng et al. administered mixed EGFR- and CD133-specific CAR-T cells for patients with advanced cholangiocarcinoma resistant to chemotherapy and radiotherapy, achieving partial responses but with acute toxicities [34]. Multi-CAR-T cells consist of two or more individual CARs targeting distinct antigens specifically expressed by each single T cell. For instance, Hedge et al. combined CARs targeting HER2 and IL-13Rα2 and demonstrated that the bispecific T cell products enhanced in vitro and in vivo CAR-T anti-tumor activity [35]. Ruella et al. generated dual anti-CD19 and anti-CD123 CAR-T cells for a strategy to treat and prevent CD19 antigen-loss escape in B-cell malignancies treated with CD19-directed CAR-T cells [36]. Following a similar approach, trivalent CAR-T cells have been engineered by expression of three CARs with different antigen specificities within the same T cells. Bielamowicz et al. used this strategy to overcome interpatient antigenic heterogeneity in glioblastoma through CAR targeting of HER2, IL13Ra2, and EphA2 [37]. Tandem CAR-T cells consist of single CAR constructs that simultaneously target different antigens through distinct antigen-binding domains. For example, Grada et al. reported TanCAR as a functional artificial bispecific molecule directed to two tumor antigens (CD19 and HER2) preserving the CAR cytolytic and antitumor activity [38]. Similarly, Hedge et al. generated tandem CAR-T cells binding to HER2 and IL13Ra2, exhibiting an enhanced T cell functionality and activity, and reducing antigen escape [39]. Other studies showed similar encouraging results using TanCAR and nanobody-based bispecific CAR-T cell strategies, for example, combining targets against CD19 and CD20 [40], CD20 and HER2 [41], or CD22 and CD19 [42] for cancer treatment.

## 3. Clinical Development of CAR-T Cell Therapies

The first clinical applications of CAR-T cells were implemented 20 years ago in patients with advanced metastatic ovarian cancer and renal cell cancer [43,44]. Kershaw et al. conducted a phase I study using gene-modified autologous T cells reactive against the ovarian-cancer-associated antigen alpha-folate receptor (FR) in combination with high-dose IL-2. In addition to this strategy, the authors also used dual-specific T cells reactive to both FR and allogenic cells, along with immunization with allogeneic peripheral blood mononuclear cells. Some of the first cohort of patients experienced grades 3 and 4 treatment-related toxicity probably due to the IL-2 administration, while the patients that received the immunization with allogeneic cells experienced grades 1 and 2 treatment-related toxicity effects. In this study, large numbers of modified tumor-reactive T cells were obtained for infusion into patients. However, these cells failed to persist in the long term and lost the ability to respond against tumor cells. In 2006, Lamers et al. described a clinical GMP protocol process to generate and expand autologous gene-modified primary human T lymphocytes for immunogene therapy for metastatic renal cell carcinoma using a carboxy anhydrase IX-specific scFv transgene [44]. In this study, functional gene-modified autologous T cells for cancer treatment were produced by retroviral transduction. In this process, T cells must be isolated from the patient or donor blood, activated, genetically modified, and expanded ex vivo to allow expression of the CAR construct. Then, the patient can be preconditioned via chemotherapy, and CAR-T cells are directly infused into the patient [17]. The following reported CAR-T clinical trials included patients with recurrent/refractory neuroblastoma [45], indolent non-Hodgkin’s follicular B-cell lymphoma, and mantle cell lymphoma [46]. In the first case, CAR-Ts were directed against the CD171 tumor antigen (L1-cell adhesion molecule), and included an expression cassette with the suicide enzyme HyTK [45]. In the second case, autologous genetically modified T cells were directed against the CD20 antigen. In both cases, persistent circulation of modified T cells after infusion and clinical efficacy were achieved, demonstrating the safety, profitability, and potential anti-cancer effect of adoptive T cell therapies. However, the major advancements will be achieved within the years to come with CAR-T cells specific to the B-lymphocyte antigen CD19 for the treatment of B-cell malignancies.

More clinical trials were conducted subsequently, mostly phase I. By the end of 2016, only 220 CAR-T cell clinical trials were being conducted. Among them, 134 were investigating CAR-T therapies for hematological malignancies (targeting mostly CD19 but also BCMA, CD123, CD138, CD16V, CD20, CD22, CD30, CD33, CD70, Ig k, IL-1RAP, Lewis Y, NKG2D ligand, and ROR1), 78 for solid tumors (targeting CEA, c-MET, EGFR, EGFRvIII, EpCAM, EphA2, ErbB2/Her2, FAP, FR-a, GD2, GPC3, IL-13Ra2, L1-CAM, Mesothelin, MUC1, MUC16ecto, PD-L1, PSCA, PSMA, and ROR1), and 9 were long-term follow-up studies [17]. At present, 1032 studies are registered at the ClinicalTrials.gov database, highlighting the relevance and avenue for improvement for these therapies in the treatment of cancer.

### 3.1. Hematological Malignancies

CAR-T cell therapy has shown significant success for the treatment of hematological malignancies, and some have been authorized by the regulatory agencies for use in different tumor types and clinical contexts.

The first two CAR-T cell therapy products targeted the B cell marker CD19 (axicabtagene ciloleucel and tisagenlecleucel) and were approved from results of relatively small non-randomized trials. After achieving 54% of complete responses in the clinical trial ZUMA-1, axicabtagene was granted approval in 2017 by the FDA and in 2018 by the EMA for the treatment of aggressive B cell lymphoma and transformed follicular lymphoma that had progressed to two lines of treatment [47]. Tisagenlecleucel was approved by the FDA and EMA for the treatment of B cell lymphoma and transformed follicular lymphoma as a consequence of the positive results from the phase II trial JULIET [48]. In this clinical trial, 40% of complete remissions were achieved in adult patients with relapsed/refractory B cell precursor acute lymphoblastic leukemia. In the same clinical context, lisocabtogene maraleucel recently received its authorization for adult patients, while patients with relapsed/refractory mantle cell lymphoma can be treated with brexucabtagene autoleucel. Lastly, patients with multiple myeloma have been able to benefit from CAR-T therapies since March 2021, as the FDA granted approval of CAR-T targeting B Cell Maturation Antigen (BCMA) after resistance or relapse to four prior lines.

Up to date, there are six CAR-T therapies approved by drug agencies worldwide (Kymriah^®^, Yescarta^®^, Tecartus^®^, Breyanzi^®^ and ARI-0001 for CAR-T against CD19+ B-cell lymphoproliferative diseases, and Abecma^®^ and Carvykti^®^ for CAR-T anti-BCMA in multiple myeloma). Tens of new proposals are under evaluation at the clinical trial level.

It must be mentioned that clinical trials of CAR-T therapies in myeloid malignancies face greater difficulties as surface antigens exclusively expressed by malignant blasts are still not known. This very challenge is one of the main limitations, but not the only one, for developing CAR-T therapies in solid tumors.

### 3.2. Solid Tumors

CAR-Ts have shown significant efficacy for the treatment of some hematological malignancies, but these therapies are largely inefficacious for the treatment of solid neoplasms. Hence, there is a major need for developing innovative CAR-T cell therapies to overcome this critical bottleneck.

**Figure 2:**
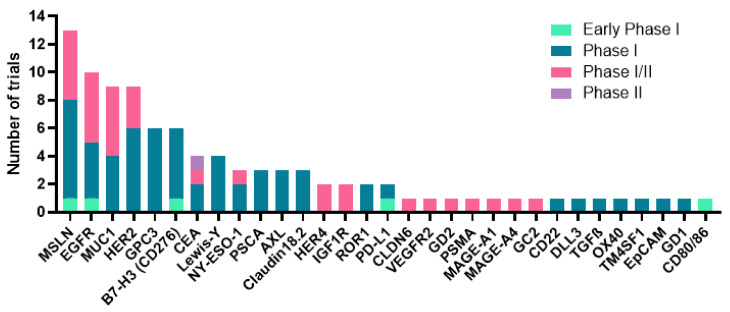
Antigens targeted in CAR-T clinical trials for lung cancer treatment. The graph represents the number of clinical trials at the indicated phases in the legend, targeting the indicated proteins.

Tumor heterogeneity has made the identification of robust and suitable targets for CAR-T cell therapy a true challenge. An additional complication arises from the need for the target antigens to be limited to tumor cells, with low or no expression in healthy tissues. Some antigens have been proposed as targets for solid tumors and are being characterized at clinical stages, including EGFR, HER2, EGFR806, Mesothelin, PSCA, MUC1, Claudin 18.2, EpCAM, GD2, AFP, Nectin4/FAP, CEA, Lewis Y, Glypican-3, EGFRvIII, IL-13Rα2, CD171, MUC16, AXL, CD20, CD80/86, c-MET, DLL-3, DR5, EpHA2, FR-α, gp100, MAGE-A1/3/4, LMP1, CAIX, MSLN, PSMA, PD-1, GPC3, ROR1, VEGFR2, or CA-125 among others [49]. The majority of these targets are being evaluated for the treatment of thoracic malignancies at various clinical stages [50] (Figure 2).

Possibly the main difficulty in the use of CAR-T cells for the treatment of solid neoplasms is the presence of a strongly immunosuppressive tumor microenvironment (TME) which is absent in hematological malignancies. The TME comprises a complex cellular and extracellular structure. Hence, it contains different cell types including leukocytes, endothelial cells, adipocytes, fibroblasts, among others. The non-cellular elements include mainly the extracellular matrix (ECM), extracellular vesicles, and cytokines [51,52]. The TME plays a major role promoting tumor invasion and metastases, and it strongly hampers the efficacy of CAR-T cells through both physical barriers and immunosuppressive soluble molecules and receptors [53].

The extracellular matrix and fibroblasts also hinder the trafficking of CAR-T cells from the bloodstream to the tumor interior. Live-cell imaging studies in tumor tissue sections have shown that active T cell motility is enhanced in regions with less fibronectin and collagen I, whereas T cells migrated poorly in dense matrix areas [54,55]. Additionally, the elevated accumulation of hyaluronan (HA) in the TME contributes to cancer progression and therapy resistance and it has been associated with lower T cell densities in the TME, supporting the idea that an HA-rich extracellular matrix can act as a shield between T cells and tumor cells preventing T cell infiltration into the TME. Even if the effector T cells succeed in infiltrating the tumor, they will face immunosuppressive activity of immune cells such as tumor-associated (M2) macrophages, myeloid-derived suppressor cells, or Tregs [56,57,58,59,60,61]. Moreover, cells composing the tumor secrete immunosuppressive factors and impede the activation of T cells or even induce their apoptosis [62,63,64,65].

Different strategies to overcome these difficulties have been proposed, including cytokine and chemokine secretion, bispecific CARs, or matrix degradation [66], as described in more detail in previous sections of this review. However, the clinical efficacies of these later generation CARs have not been formally evaluated yet in clinical trials.

### 3.3. Current Development of CAR-T Clinical Trials for Lung Cancer

**Figure 3:**
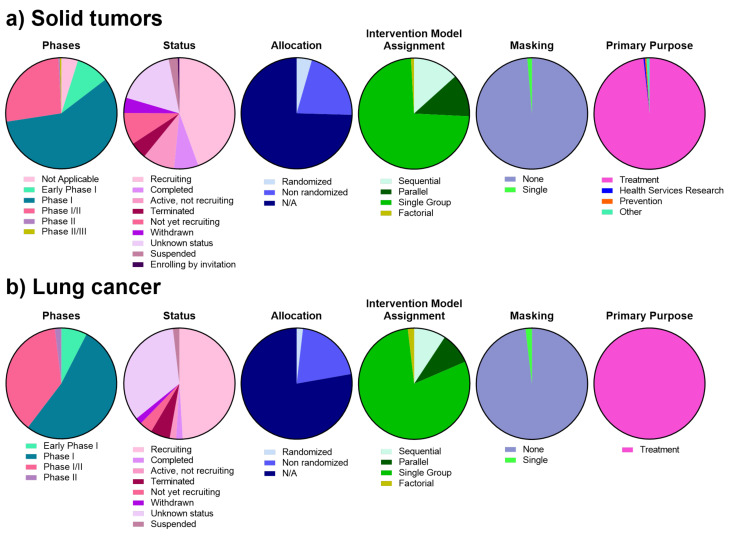
Categorization of CAR-T cell interventional clinical trials for lung cancer on phases, studied conditions, status, and study design (allocation, intervention model assignment, masking, and primary purpose). (**a**) Interventional clinical trials studying CAR-T for solid tumors. (**b**) Interventional clinical trials studying CAR-T for lung cancer.

Currently, more than 200 interventional clinical trials are evaluating the efficacy of CAR-T therapies in solid tumors (Figure 3a). Most of them are phase I and phase I/II, open label, and single group assignment clinical trials. The solid tumors targeted in these clinical trials are mainly liver, peritoneal, colon, colorectal, pancreatic, gastric, biliary tract, renal, lung, ovarian, fallopian tube, melanoma, breast, glioblastoma, neuroblastoma, cervical, sarcoma, osteosarcoma, thyroid, prostate, nasopharyngeal, retinoblastoma, and medullary cancers. Only one clinical trial has reached phase II/III for the treatment of pancreatic cancer with liver metastases, although recruitment has not started yet (NCT04037241).

At present, 50 interventional clinical trials are studying CAR-T in lung cancer patients (Figure 3b) (Supplementary Materials Table S1). Most of them are phase I and non-comparative phase I/II basket trials and include heterogeneous cohorts of patients diagnosed with different tumor types that share the expression of a common surface protein. Only a minority of CAR-T clinical trials in non-small cell lung cancer (NSCLC) patients have reported preliminary results, describing mostly modest clinical activities. An early clinical trial with an MSLN-targeted CAR in MSLN (Mesothelin)-expressing solid tumors was interrupted before reaching phase II as no responses were observed among 15 patients (NCT01583686). Another trial with a second-generation PD-L1-targeted CAR was also interrupted because the first patient experienced severe cytokine release syndrome (CRS) causing interstitial pneumonia that required high-dose corticosteroids and tocilizumab administration under intensive care (NCT03330834). A second-generation anti-ROR1 CAR-T construct (NCT02706392) induced a mixed response in two NSCLC patients with no relevant toxicity besides grade 1 CRS [67,68]. Promising preliminary results from a phase I clinical trial using CLDN6-targeted CAR-T cells, combined or not with a CAR-T cell amplifying RNA vaccine, were presented in ESMO Immuno-Oncology Congress 2021 reporting tumor shrinkage in 3 out of 5 patients [69]. A phase I trial of MUC1-targeted CAR-T cells with PD-1 knockout through CRISPR-Cas9 for the treatment of NSCLC patients (NCT03525782) had manageable toxicity and no grade 3–5 adverse events. However, no clear signs of activity were detected. From the 20 patients enrolled in the study, 11 (55%) experienced stable disease as best response, while 9 (45%) progressed after treatment [70]. Four HLA-A2-positive patients with metastatic NY-ESO-1 positive NSCLC received NY-ESO-1-targeted CAR-T cells along with IL-2. One experienced stable disease for 3 months, another had a partial response that lasted 4 months, and the remaining two progressed shortly after treatment. Tolerance, however, was acceptable, and only one patient presented a G3 adverse event [71]. Disease stabilization at 4 weeks was achieved in one patient treated with a tnMUC1-targeted CAR co-expressing the CD2 and CD3ζ intracellular signaling domains. In this study, improved CAR expansion was observed after a previous lymphodepleting chemotherapy had been administered [72]. Lastly, among nine patients with EGFR-positive NSCLC receiving a second-generation EGFR-targeted CAR-T, one had a radiological response that persisted for more than 1 year, with squamous NSCLC, and two experienced disease stabilization for 2 months. The proportion of central memory and effector memory CAR-T cells was 23.5% and 25.8% within the patients [73].

Currently, 53 tumor specific antigens are being targeted in CAR-T cell clinical trials for lung cancer in 4 early phase I, 28 phase I, 20 phase I/II, and 1 phase II clinical trials (Figure 3). One of the most prevalent targets in these trials is MUC1. The aberrant overexpression of mucins is associated with cancer cell growth and metastasis. Mucin 1 (MUC1) is the most frequently targeted antigen in lung cancer clinical trials, especially for the treatment of NSCLC. MUC1 is a cell membrane glycoprotein overexpressed in human lung cancer, amongst others. It has been described to have a role in carcinogenesis and progression from premalignant lung lesions to invasive carcinoma [74,75]. Mesothelin is possibly the second most targeted antigen in lung cancer CAR-T clinical trials. Mesothelin is expressed in advanced stage lung adenocarcinomas and is associated with inferior survival [76]. A recent potential target for CAR-T therapies in NSCLC is PD-L1. This molecule is a well-recognized biomarker for the efficacy of PD-L1/PD-1 immunotherapies. Indeed, PD-1 axis blockers have been established as an effective first line of treatment [4,5,7,77,78,79,80,81,82,83,84,85,86]. PD-1 and PD-L1 are being targeted on CAR-T clinical trials for lung cancer through αPD1-MSLN-CAR-T cells (NCT04489862), Zeushield Cytotoxic T Lymphocytes (NCT03060343), anti-CTLA-4/PD-1 expressing CAR-Ts (NCT03182816, NCT03182803), HerinCAR-PD1 cells (NCT02862028, NCT04429451), combination products (NCT03525782), or autologous aPD-L1 armored anti-CD22 CAR-T cells (NCT04556669). In fact, many CAR-T cells targeting the PD-1/PD-L1 axis are being developed at a preclinical level, showing promising in vitro and in vivo antigen-specific activation, cytokine production, and effective suppression of tumor growth [87,88,89,90]. CTLA-4 expression in NSCLC might be relevant as well, although without a clear prognostic value [91]. Some phase I/II clinical trials are investigating anti-CTLA-4/PD-1 expressing EGFR-CAR-Ts or expressing mesoCAR-Ts. Furthermore, HER2 has demonstrated to play a role in NSCLC, as several mutations and aberrations in *HER2* have been described in NSCLC patients. Hence, particularly among the *HER2* exon 20 mutations, mutant HER2 remains a potential therapeutic target for NSCLC treatment, as well as a prognostic factor [92]. In addition, more than 60% of NSCLC carcinomas express EGFR, being the main NSCLC tumor driver when activating mutations are present [93,94]. Thus, EGFR has a relevant role in NSCLC tumor progression and treatment resistance [94,95,96]. GPC3 protein expression also showed clinical significance in lung squamous cell carcinoma and lung adenocarcinoma [97]. In fact, lung cancer patients with metastasis and with poorly differentiated cancer exhibited an increase in GPC-3 expression, with an impact on prognosis [97,98]. CD276 (or B7-H3) is also being targeted in the lung cancer CAR-T clinical landscape. Indeed, it is a new promising co-inhibitory checkpoint molecule with an important role in the regulation of T cell responses [99,100]. B7-H3 is expressed in NSCLC, and it is associated with tumor progression and metastasis, tumor immune evasion, and infiltrating FOXP3+ Tregs [101,102]. TM4SF1 is also upregulated in lung cancer, both in cell lines and tissues [103]. The expression of this protein is associated with lung cancer progression, tumor cell growth, treatment resistance, invasion, and metastasis. Other relevant antigens that have been proposed as targets include CLDN6, GD2, CEA, PSMA, NY-ESO1, Lewis-Y, and PD-L1. Overexpression of these antigens are associated with worse prognosis, cell proliferation, and migration [71,104,105,106,107,108,109,110,111]. For instance, GD2 is highly expressed in SCLC cells [105,112], PSMA and NY-ESO1 are expressed in NSCLC and SCLC tumor cells, among other tumor types [108,109,113]. NSCLC patients with tumors expressing NY-ESO-1 exhibited clinical responses to NY-ESO1-specific TCR T cell therapy [71]. Finally, VEGFR2, MAGE-A1, MAGE-A4, PSCA, AXL, Claudin 18.2, TGFB, CD22, ROR1, OX40, DLL3, MSLN, and EpCAM are being targeted in CAR-T cell clinical trials for lung cancer. All of them are differentially expressed in lung cancer, more commonly in NSCLC, and correlate with tumor cell proliferation, treatment resistance, and poor prognosis [104,114,115,116,117,118,119,120,121,122,123,124,125,126,127,128,129,130,131,132,133,134,135,136,137]. Thus, there is robust evidence supporting the utility of all these tumor specific antigens as emerging targets and tumor markers for the development of CAR-T cell-based therapeutic strategies for the clinical development of novel immunotherapies for lung cancer and other solid tumors.

## 4. Conclusions

The use of CAR-T cell technology for the treatment of solid tumors has turned out to be very challenging in numerous clinical trials, with only very limited success. The high cost and the considerable amount of time required for the production of CAR-T cell therapies, with the risk of losing the window of opportunity to treat the patient due to clinical deterioration, are barriers faced both by patients with hematological neoplasms and solid tumors. Moreover, functionality and persistence of CAR-T cells generated from lymphocytes of heavily treated patients can be difficult due to genotoxic damage. The ongoing development of universal CAR-T generated from allogenic donors might overcome these limitations, although this approach carries its own challenges.

Regarding the treatment of solid tumors, the identification of suitable specific target antigens for CAR-T therapies is more difficult than for hematological neoplasms. However, with the support of public databases such as “The Cancer Genome Atlas” (TCGA), providing genomic and proteomic data of thousands of tumor samples covering different cancer types, a significant effort has been invested to identify and test target antigens which could have significant therapeutic utility, together with increasing efficacy, survival, persistence, and safety of CAR-T therapies specifically for lung cancer. The TME is also a challenge restricted to the treatment of solid tumors. It plays a key role preventing the infiltration and antitumor response of CAR-T cells through physical barriers and the immunosuppressive activities of both tumor and host cells. Novel approaches are needed to either enhance the design of CAR constructs or complement these CAR-T cells with strategies to overcome the TME. The modification of the TME with different strategies including matrix degradation, immune-checkpoint local blockade with antibody/nanobody producing CAR-T cells, or immunosuppressive cell depletion might be key for improving the efficacy.

To sum up, the development of innovative CAR molecular engineering strategies with the potential to deliver effective and long-lasting tumor control will be one of the most promising advancements for the treatment of lung cancer. The preclinical and clinical development of new approaches that achieve better CAR-T cell infiltration and survival within the microenvironment of solid cancers in solid tumors, and particularly in lung cancer, is of vital importance to successfully treat solid tumors.

## Supplementary Materials

The following supporting information can be downloaded at: https://www.mdpi.com/article/10.3390/life12040561/s1, Table S1: Clinical trials evaluating CAR-T cells in patients with lung cancer.
